# Association between the mediterranean diet and cognitive health among healthy adults: A systematic review and meta-analysis

**DOI:** 10.3389/fnut.2022.946361

**Published:** 2022-07-28

**Authors:** Jialei Fu, Li-Juan Tan, Jung Eun Lee, Sangah Shin

**Affiliations:** ^1^Department of Food and Nutrition, Chung-Ang University, Seoul, South Korea; ^2^Department of Food and Nutrition, College of Human Ecology, Seoul National University, Seoul, South Korea

**Keywords:** cognitive function, mild cognitive impairment, dementia, Alzheiemer’s disease, Mediterranean diet (MD)

## Abstract

**Background:**

An increasing prevalence of cognitive disorders warrants comprehensive systematic reviews on the effect of diet on cognitive health. Studies have suggested that the Mediterranean (MeDi) diet has protective effects against metabolic diseases. However, comprehensive systematic reviews on the effect of the MeDi diet on the cognitive decline are limited. We investigated whether adherence to the MeDi diet could lower the risk of the cognitive disorder or improve cognitive function in older adults.

**Methods:**

In this systematic review and meta-analysis, PubMed, Web of Science, PsycINFO, Scopus, and Cochrane databases were searched from inception to June 2021. Cohort studies and randomized controlled trials (RCTs) were included. The effect sizes were estimated as log risk ratios and standard mean differences (SMDs) with 95% confidence intervals (CIs). The Newcastle–Ottawa score and Cochrane Collaboration’s tool were used to assess the risk of bias in cohort studies and RCTs, respectively.

**Results:**

Of the 1,687 screened studies, 31 cohort studies and five RCTs met the eligibility criteria for qualitative analysis; 26 cohort studies and two RCTs were included in the meta-analysis. In the cohort studies, high adherence to the MeDi diet was associated with lower risk of mild cognitive impairment (MCI) [risk ratio (RR) = 0.75 (0.66–0.86)], and Alzheimer’s disease (AD) [RR = 0.71 (0.56–0.89)]. In the RCTs, high adherence to the MeDi diet was associated with better episodic [SMD = 0.20 (0.09–0.30)] and working memories [SMD = 0.17 (0.01–0.32)] than lowest group.

**Conclusion:**

Adherence to the MeDi diet may reduce the risk of MCI and AD. However, other associations with cognitive outcomes (global cognition, working memory, and episodic memory) remain open to interpretation. Overall, the MeDi diet is recommended to prevent or delay cognitive disorders and improve cognitive function. Further, long-term RCTs are warranted to strengthen the evidence.

**Systematic review registration:**

[https://www.crd.york.ac.uk], identifier [CRD42021276801].

## Introduction

Mild cognitive impairment (MCI) is defined as a cognitive decline greater than that expected for the age and education level of the individual while not interfering with activities of daily living (1). MCI is a stage in the progression from normal cognitive function to dementia (2). Globally, dementia is the seventh most common cause of death and the most common cause of illness in older adults. According to the WHO, there are currently more than 55 million confirmed cases of dementia worldwide, and the number of new cases is increasing at a rate of 10 million per year. In addition to this, the number of people with dementia is projected to grow to 78 million by 2030 and 139 million by 2050. This is owing to the increasing proportion of older people worldwide. The most commonly diagnosed form of dementia is Alzheimer’s disease (AD), which accounts for approximately 60–70% of cases (3). According to the Alzheimer’s Association, the number of deaths from AD increased by 145% from 2000 to 2019 (4). Moreover, during the coronavirus disease pandemic, deaths owing to AD and dementia have increased by 16% (5). AD is an irreversible degenerative brain disease (6), and currently, there is no cure for dementia (3). Therefore, determining whether cognitive impairment can be prevented or delayed by dietary modification is important.

The Mediterranean (MeDi) diet is a dietary pattern that has been followed by the Mediterranean Basin countries since the early 1960s (7) and is mainly based on abundant plant-based consumption with food that is minimally processed, seasonal, fresh, and locally grown. Fresh fruit is consumed every day, and olive oil is the main source of fat. Additionally, low-to-moderate amounts of fish and seafood, poultry, and dairy products are consumed daily. A regular but moderate amount of wine is also consumed, along with 0–4 eggs that are consumed per week. Sweets containing sugar or honey and red meat are sparingly consumed (8).

The association between the MeDi diet and increased longevity and reduced mortality and morbidity from certain cancers and other nutrition-related diseases has been widely studied (9–12). However, whether adherence to the MeDi diet can prevent or delay the risk of cognitive disorders and improve cognitive function remains understudied. While some epidemiological studies did not show a relationship (13–15), others have shown positive associations between the MeDi diet and the prevention of cognitive disorders and improvement in cognitive function (16–19). Although several systematic reviews focusing on cognitive disorders or cognitive function have been published (20–25), there is no systematic review to quantitatively evaluate the association between cognitive disorders and cognitive function from both prospective studies and RCTs simultaneously. In addition, although previous reviews conducted meta-analyses, they did not conduct any further analysis to investigate the high heterogeneity source, which might lead to results bias. Therefore, we performed a systematic review and meta-analysis of both cohort studies and randomized controlled trials (RCTs) to comprehensively analyze the association between adherence to the MeDi diet and cognitive disorders (i.e., MCI, dementia, and AD) and cognitive functions (i.e., attention, episodic memory, executive function, global cognition, processing speed, and working memory). Furthermore, we conducted subgroup and meta-regression analyses to identify whether a wide range of characteristics contributed to the differences in the results of the cohort studies.

## Methods

### Literature search

This systematic review and meta-analysis followed the Preferred Reporting Items for Systematic Reviews and Meta-Analyses (PRISMA) guidelines (26). A protocol was designed and registered in the PROSPERO database (PROSPERO 2021 CRD42021276801). We searched five electronic databases: PubMed, Web of Science, PsycINFO, Scopus, and Cochrane, for articles published before June 2021. The search terms used were “Mediterranean diet,” “mild cognitive impairment,” “dementia,” “Alzheimer’s disease,” and “cognitive function,” and only English articles were included. Two authors independently screened the titles and abstracts of the research papers. After this, they read the full texts to identify potentially eligible studies. The PICOS criteria are shown in Table 1; see Supplementary Table 1 for the detailed search methodology.

**TABLE 1:** PICOS criteria for inclusion of studies.

Parameter	Description
Population	Cognitively normal adults
Intervention	Adherence to the MeDi diet
Comparison	Normal diet or low adherence to the MeDi diet
Outcomes	Mild cognitive impairment or dementia or Alzheimer’s disease or cognitive function
Study design	Cohort studies or RCTs

RCT, randomized controlled trial; MeDi diet, Mediterranean diet.

### Selection criteria

Studies were selected and included based on the following inclusion criteria:

1. Studies that were published in English, with no restrictions on the study sample size or the participants’ age, sex, or health status.
2. Studies that were RCTs or prospective observational studies and investigated the relationship between adherence to the MeDi diet and cognitive function (including attention, episodic memory, executive function, global cognition, processing speed, or working memory) or the risk of cognitive disorders (including MCI, dementia, or AD).
3. If two studies used the same cohort database, both were included.

The exclusion criteria were as follows:

1. Studies that did not adhere to the MeDi diet. For example, studies with adherence to a particular national dietary pattern or a healthy eating index.
2. Studies that included participants with cognitive disorders or abnormal cognitive functioning at baseline.
3. Case-control studies, cross-sectional studies, systematic reviews, narrative reviews, conference reports, or letters.

### Data extraction

Two authors (JF and L-JT) independently extracted data using the same extraction method. In the case of a dispute, a third author (SS) helped reach a consensus. The data on the last name of the first author, publication year, the health status of participants, follow-up duration, baseline age, percentage of men, sample size, dietary assessment method, MeDi diet assessment method, cognitive assessment methods, cognitive domains measured, and results were extracted from cohort studies. For the RCTs, data on the last name of the first author, publication year, country, population selection, follow-up duration, intervention group, placebo group, dietary assessment methods, and the MeDi diet assessment method, the baseline age and number of participants, outcome test methods, and results were extracted. Two reviewers independently assessed cognitive disorders and cognitive function of cohort studies and cognitive function of RCTs. Cognitive disorders included MCI, dementia, and AD, which were based on the battery of neuropsychological test or diagnosis criteria. Cognitive function included attention, episodic memory, executive function, global cognition, processing speed, and working memory, which were tested by the Mini-Mental State Examination (MMSE) or other common cognitive function tests [e.g., Telephone Interview for Cognitive Status (TICS), the Digit Span-Backward Test (DST)].

### Quality assessment

Two independent reviewers evaluated the quality of the cohort studies and RCTs. The Newcastle–Ottawa score (NOS) was used to evaluate the quality of the prospective cohort studies (27). Scores of ≥ 7 were considered high-quality scores, and scores of ≤ 4 were considered low-quality scores. The Cochrane Collaboration tool was used to evaluate the risk of bias in RCTs (28). The deviation from risk assessment criteria for each factor was divided into three levels—“high,” “low,” and “unclear” risk of bias.

### Statistical analyses

We performed a meta-analysis on three cognitive disorders and six cognitive domains. From the cohort studies, we extracted risk ratios (RRs) [hazard ratio (HRs) or odds ratio (ORs)] with 95% confidence intervals (CIs) for cognitive disorders (MCI, dementia, and AD) and effect sizes and standard errors for cognitive function (episodic memory, global cognition, and working memory). Owing to the asymmetry of the RRs, these values were log-transformed (base 10) (29). From RCTs, we extracted changed means and changed standard deviation (SD) for cognitive function (attention, episodic memory, executive function, global cognition, processing speed, and working memory). If the results of the original manuscript contained only the means or SD of the baseline and final groups, the changed mean was calculated by subtracting the final mean from the baseline mean. Changed SD was obtained using the following equation, where the correlation coefficient R was set at 0.5 (30).


$$S\,D_{c\,h\,a\,n\,g\,e}=\sqrt{S\,D_{b\,a\,s\,e\,l\,i\,n\,e}^{2}+S\,D_{f\,i\,n\,a\,l}^{2}-2\times R\times S\,D_{b\,a\,s\,e\,l\,i\,n\,e}\times S\,D_{f\,i\,n\,a\,l}}$$

If the study reported 95% CI instead of SD, we used the following formula:


$$S\,D=\sqrt{N}\times \frac{95\%\,C\,I_{u\,p\,p\,e\,r}-95\%\,C\,I_{l\,o\,w\,e\,r}}{2\times t\,v\,a\,l\,u\,e}$$

All statistical analyses were performed in STATA MP 17.0 and the Review Manager 5.4. A two-tailed *P* value of < 0.05 was considered statistically significant. The heterogeneity test in this review was examined using the Cochran’s Q test and quantified using the I^2^ statistic. For medium and low heterogeneity (I^2^ of < 50%), fixed-effects models were used, while random-effects models were used for high heterogeneity (I^2^ of ≥ 75%) (31, 32). Moreover, potential sources of heterogeneity were examined using meta-regression and subgroup analysis based on covariates such as study location: (1) Mediterranean region or (2) non-Mediterranean region; publication year: (1) after 2015 or (2) before 2015; duration of follow-up: (1) ≥ 5 years or (2) < 5 years; method of assessing dietary intake: (1) food frequency questionnaire (FFQ) or (2) others; and study quality: (1) scores = 9, (2) scores = 8, or (3) scores = 7 (30, 33, 34). Publication bias was assessed based on at least 10 studies and was quantitatively assessed using Egger’s test, Begg’s test, and funnel plots (35).

## Results

### Literature selection

**FIGURE 1:**
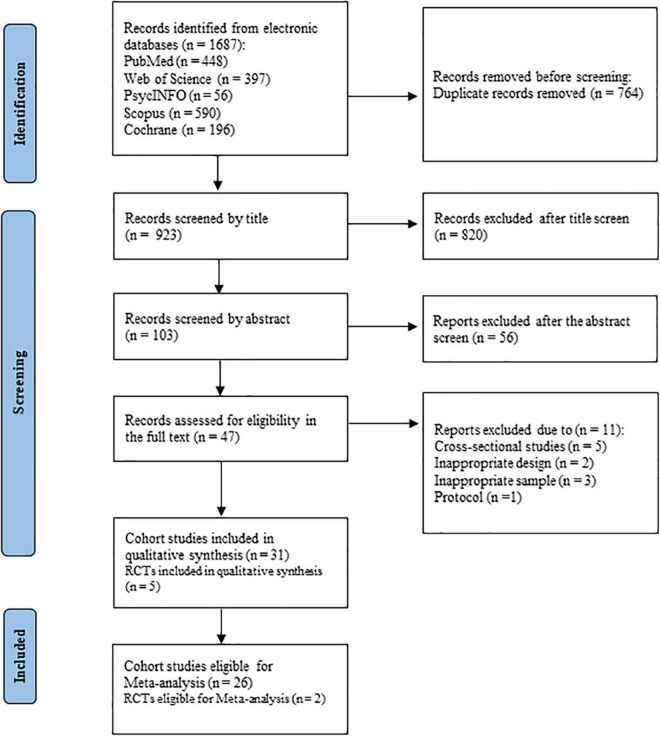
Flow chart of study identification and selection.

A total of 1,687 studies in PubMed, Web of Science, PsycINFO, Scopus, and Cochrane databases were identified. Of these, 820 studies were excluded after searching the keywords and reviewing the studies by their titles to assess relevance and eligibility. The abstracts of 103 studies were reviewed, after which the full texts of 47 studies were assessed. After excluding irrelevant studies and including reviews and cross-sectional studies, 31 cohort studies and five RCTs were included in the qualitative assessment. Finally, 26 cohort studies and two RCTs that met the criteria were included in the meta-analysis (Figure 1).

### Study characteristics

In total, 31 cohort studies and five RCTs were included in the review. Characteristics of prospective studies and RCTs are summarized in Tables 2 and 3, respectively. Nine cohort studies were conducted in the Mediterranean countries of France, Greece, Spain, and Italy (15, 36–43), while the other 22 studies were from China, the United States of America, Australia, Sweden, the United Kingdom, and Singapore (13, 14, 16–19, 44–60). Most studies (*n* = 25) were women dominant, with five studies including only women and three studies including only men (19, 41, 47). The sample sizes varied from 200 to 27,842 participants. Additionally, follow-up median or mean durations ranged from 2.2 to 26 years, and baseline ages ranged from 45 to 92 years. The cut-off value selected by most of the studies was over 65 years (*n* = 13). The majority of studies used an FFQ to assess the MeDi diet (*n* = 26), and two of these studies used both an FFQ and a 24-h dietary recall to assess diets (15, 16). Among the studies using an FFQ, two used the Council of Victoria FFQ and Women’s Health Initiative FFQ, while the others used the food groups’ semi-quantitative FFQ (14, 45). Moreover, the MeDi diet was primarily scored on a 0–9 scoring range (*n* = 25), while 1 study used a 0–15 MeDi diet score, defined by Morris et al. (55, 61) and 5 studies used a 0–55 MeDi diet score, defined by Panagiotakos et al. (17, 36, 46, 56, 57). Cognitive function was assessed using a large number of tests to quantify the cognitive domains of global cognition, episodic memory, working memory, processing speed, executive function, and attention. The most common and widely used test was MMSE (*n* = 19), which included tests on attention, language, memory, visual-spatial skills, and orientation. Owing to individual differences in research, studies used different cognitive function tests, including the DST, TICS, Benton Visual Retention Test (BVRT).

**TABLE 2:** Main characteristics of the included cohort studies.

References	Country	Study characteristics and demographics					Mediterranean diet score		Cognitive outcome measure		Results
		Participant selection	Follow-up, y	Baseline age	Male (%)	Sample size	Diet method	MeDi score	Method	Cognitive domains	
Bhushan et al. (19)	United States	US male Health professionals	26	40–75	100	27,842	FFQ	0–9 MeDi diet score	SCF score	Global cognition	The MeDi diet was strongly related to lower subjective cognitive function
Charisis et al. (36)	Greece	Non-demented individuals	3	64	40.3	1,046	FFQ	0–55 MeDi diet score	NT	Memory, language, attention, executive function, visuospatial perception	The MeDi diet was associated with a reduced risk for dementia and cognitive decline
Cherbuin and Anstey (13)	Australia	Healthy participants	4	60–64	48.2	1,528	FFQ	0–9 MeDi diet score	MMSE	Global cognition	The MeDi diet was not found to be protective against cognitive decline
Feart et al. (15)	France	Healthy participants without dementia	4.1	>65	37.4	1,410	FFQ and 24 h dietary recall	0–9 MeDi diet score	MMSE, IST, BVRT, FCSRT	Global cognition, semantic verbal fluency, verbal production speed, immediate visual memory, verbal episodic memory	The MeDi diet was associated with lower MMSE cognitive decline
Galbete et al. (39)	Spain	Healthy Spanish	8	>55	71.0	823	FFQ	0–9 MeDi diet score	TICS-m	Immediate memory, delayed recall, orientation, attention, calculation, language	High adherence to the MeDi diet might be associated with better cognitive function
Gallucci et al. (40)	Italy	Healthy participants	7	>77	38.8	309	FFQ	0–9 MeDi diet score	MMSE	Global cognition	There was no significant association between the MeDi diet and cognitive function
Gardener et al. (45)	Australia	Healthy participants	3	>60	39.8	527	CCVFFQ	0–9 MeDi diet score	Global cognitive score	Verbal memory, visual memory, executive function, language, attention, visuospatial function	The AusMeDi diet was associated with better performance in the executive function cognitive domain
Gu et al. (18)	United States	Healthy participants without dementia	3.8	>65	33.4	1,219	FFQ	0–9 MeDi diet score	Composite cognitive Z-score	Memory, language, processing speed, visual-spatial ability	Better adherence to the MeDi diet was significantly associated with lower risk for AD
Haring et al. (14)	United States	Healthy participants without dementia	9.11	65–79	0	6,425	WHI-FFQ	0–9 MeDi diet score	MMSE, CERAD, DSM-IV	Global cognition, verbal fluency, verbal learning and memory, constructive praxis, executive function	Adherence to the MeDi diet did not modify the risk for cognitive decline
Kesse-Guyot et al. (42)	France	Healthy participants	13	>45	53.7	3,083	24 h dietary recall	0–9 MeDi diet score	RI-48, VFT, DST, Delis-Kaplan TMT	Episodic memory, semantic memory, short-term and working memory, mental flexibility	There was no beneficial effect of the MeDi diet adherence on cognitive function
Koyama et al. (46)	United States	Healthy participants	7.9	70–79	48.7	2,326	FFQ	0–55 MeDi diet score	3MS score	Orientation, registration, attention, recall, visuospatial ability	High adherence to the MeDi diet may reduce the rate of cognitive decline among black older adults, but not white older adults
Lutski et al. (41)	Israel	Cognitively normal participants	4.8	45–74	100	200	4-day dietary record	0–9 MeDi diet score	Computerized cognitive test	Memory, executive function, visual-spatial, attention	Poor vs high adherence was associated with a greater decline in overall cognitive performance
Morris et al. (17)	United States	Healthy old adults without AD	4.5	58–98	24.0	923	FFQ	0–55 MeDi diet score	Alzheimer’s incidence rate	Episodic memory, global cognition, processing speed, reasoning, semantic memory, working memory	The MeDi diet was associated with lower AD rates
Olsson et al. (47)	Sweden	Healthy participants	12	>70	100	1,038	7-day dietary record	0–9 MeDi diet score	MMSE, NINCDS-ADRDA, DSM-IV	Global cognition	Adherence to the MeDi diet did not modify the risk for cognitive decline
Psaltopoulou et al. (43)	Greece	Healthy participants	8	>60	35.1	732	FFQ	0–9 MeDi diet score	MMSE, GDS score	Global cognition	PUFA and seed oil as two dietary variables which were alternatively substituted for MeDi diet Score may have adverse effects on performance on cognitive function
Qin et al. (48)	China	Healthy participants	5.3	>55	49.7	1,650	24 h dietary recall	0–9 MeDi diet score	Cognitive screening test	Immediate memory, orientation	There was no association among adults aged < 65 years, among adults aged > 65 years, adherence to the MeDi diet had a slower rate of cognitive decline
Roberts et al. (49)	United States	Healthy participants	2.2	70–89	53.0	1,233	FFQ	0–9 MeDi diet score	CDR, NT	Memory, executive function, language, visuospatial	Adherence to the MeDi diet did not modify the risk for cognitive decline
Samieri et al. (50)	United States	Healthy older women	6	>70	0	16,058	FFQ	0–9 MeDi diet score	TICS, EBMT, TICS-m, category fluency test, DST	Global cognition, verbal memory, working memory, attention	Long-term MeDi diet adherence was related to moderately better cognitive change
Samieri et al. (51)	United States	Healthy older women	4	>65	0	6,174	FFQ	0–9 MeDi diet score	TICS, EBMT, TICS-m, category fluency test, DST	Global cognition, verbal memory	No association of the MeDi diet with cognitive decline
Scarmeas et al. (52)	United States	Cognitively normal participants	4.5	>65	32.0	1,393	FFQ	0–9 MeDi diet score	Alzheimer’s incidence rate, CDR	Memory, executive, language, visuospatial	Higher adherence to the MeDi diet was associated with a reduced risk of developing MCI
Scarmeas et al. (53)	United States	Cognitively normal participants	4.3	>65	31.0	1,880	FFQ	0–9 MeDi diet score	NAB	Memory, orientation, language, construction	Higher adherence to the MeDi diet was associated with a reduced risk of developing AD
Scarmeas et al. (54)	United States	Cognitively normal participants	4	>65	32.0	2,258	FFQ	0–9 MeDi diet score	NAB	Memory, orientation, language, construction	Higher adherence to the MeDi diet was associated with a reduced risk of developing AD
Shannon et al. (55)	United Kingdom	Healthy older individuals with CVD risk	13	48–92	44.0	8,009	FFQ	0–15 MeDi diet score	SF-EMSE, HVLT	Global cognition, verbal episodic memory, nonverbal episodic memory, attention, simple processing speed, complex processing speed, memory	High adherence to the MeDi diet was associated with good cognitive function and low risk of poor cognition in older adults: verbal episodic memory
Tanaka et al. (37)	Italy	Cognitively normal participants	10.1	>65	43.5	832	FFQ	0–9 MeDi diet score	MMSE	Global cognition	Adherence to the MeDi diet can have long-lasting protective effects on cognitive decline and may be an effective strategy to prevent or delay dementia
Tangney et al. (56)	United States	Healthy participants	4.1	>65	26.0	826	FFQ	0–55 MeDi diet score	19 cognitive tests	Global cognition, episodic memory, executive function, processing speed, semantic memory, working memory	The MeDi diet pattern may reduce the rate of global cognitive decline with older age
Tangney et al. (57)	United States	Healthy participants	7.6	>65	38.3	3,790	FFQ	0–55 MeDi diet score	EBMT, MMSE, SDMT	Global cognition	The MeDi diet pattern may reduce the rate of cognitive decline with older age
Trichopoulou et al. (38)	Greece	Healthy participants	6.6	>65	35.9	401	FFQ	0–9 MeDi diet score	MMSE	Global cognition	Adherence to the traditional MeDi diet was highly likely to protect against cognitive decline
Tsivgoulis et al. (58)	United States	Participants without MCI	4	45–98	43.0	17,478	FFQ	0–9 MeDi diet score	SIS	Cognition	Adherence to the MeDi diet was associated with a lower likelihood of ICI in nondiabetic participants
Vercambre et al. (59)	United States	US female Health professionals	5.4	>65	0	2,504	FFQ	0–9 MeDi diet score	TICS, EBMT, TICS-m, EBMT	Global cognition, verbal memory, category fluency	No association of the MeDi diet with subsequent 5-year cognitive change
Wengreen et al. (60)	United States	Cognitively normal participants	10.6	>65	42.9	3,580	FFQ	0–9 MeDi diet score	3MS score	Global cognition	Adherence to the MeDi diet was associated with cognitive function in older men and women
Wu et al. (16)	Singapore	Healthy participants	19.7	45–74	40.8	16,948	FFQ and 24 h dietary recall	0–9 MeDi diet score	SM-MMSE	Global cognition	Adherence to the MeDi diet patterns in midlife is associated with a lower risk of cognitive impairment in later life in Chinese adults

MeDi diet, Mediterranean diet; SCF, Subjective cognitive function; NT, neuropsychological test; MMSE, Mini-Mental State Examination; IST, Isaacs set test; BVRT, Benton visual retention test; FCSRT, Free and cued selective reminding test; TICS-m, Telephone interview of cognitive status-modified; CCVFFQ, Council of Victoria food frequency questionnaire; AusMeDi diet, Australia Mediterranean diet; WHI-FFQ, Women’s health initiative food frequency questionnaire; CERAD, Consortium to establish a registry for Alzheimer’s disease; DSM-IV, Diagnostic and statistical manual of mental disorders; RI-48, Rappel Indicé (cued recall)-48 items; TMT, trail-making test; VFT, verbal fluency tasks; DST, Digit span-backward test; 3MS score, Modified Mini-Mental State Examination score; SES, Socioeconomic status; NINCDS-ADRDA, National institute of neurological and communication disorders and stroke-Alzheimer’s disease; GDS score, Geriatric depression scale score; TICS, Telephone interview for cognitive status; EBMT, East Boston memory test; PUFA, Polyunsaturated acids; CDR, Clinical dementia rating; MCI, Mild cognitive impairment; AD, Alzheimer’s disease; NAB, Neuropsychological Assessment Battery; SF-EMSE, Short form extended mental state exam; SDMT, symbol digit modalities test; HVLT, Hopkins verbal learning test; SIS, The six-items screener; ICI, Incident cognitive impairment; SM-MMSE, Singapore modified MMSE.

**TABLE 3:** Main characteristics of the included randomized controlled trials.

References	Country	Population	Duration	Intervention	Placebo group	Dietary	MeDi score	Baseline age	Subjects (*N*)	Loss	Outcome method	Results
Hardman et al. (62)	Australia	Healthy older adults	0.5 years	The diet group received a collection of recipes in keeping with MeDi diet style, and with the assistance of dietary consultants from Health Care 2	Maintenance of their current lifestyle	FFQ	0–9	EG: 77.68 ± 7.38 CG: 78.22 ± 5.81	EG: 18 CG: 25	EG: 28% CG: 7%	SUCCAB	The MeDi diet has the potential to improve aspects of cognition in aging population
Knight et al. (63)	Australia	Healthy older adults	0.5 years	Participants were closely monitored on a fortnightly basis in an informed meeting that followed MeDi diet food pyramids	Participants were asked to simply maintain their customary lifestyle and dietary pattern	FFQ	0–9	EG: 72.1 ± 4.9 CG: 72.0 ± 5.0	EG: 70 CG: 67	EG: 19% CG: 17%	A comprehensive battery of 11 cognitive tests	There was no beneficial effect of the MeDi diet intervention on cognitive function among healthy older adults
Martinez-Lapiscina et al. (64)	Spain	Cognitively normal participants at high CVD risk	6.5 years	Participants received intensive education and advice to increase adherence to MeDi diet. Participants received free allotments of either EVOO (1 L/week) or 30 g/day of raw, unprocessed mixed nuts (15 g walnuts, 7.5 g almonds and 7.5 g hazelnuts)	Participants received intensive education and advice to increase adherence to the low-fat diet and received advice to reduce all types of fat and non-food gifts as an incentive to improve compliance	FFQ	0–14	All: 67.38 ± 5.65	EGE: 224 EGN: 166 CG: 132	EGE: 36% EGN: 53% CG: 63%	MMSE, CDT	An intervention with the MeDi diet enhanced with either EVOO or nuts appears to improve cognition compared with a low-fat diet
Sanchez-Villegas et al. (65)	Spain	Cognitively normal participants at high CVD risk	3 years	The groups assigned to MeDi diet were advised to use extra virgin olive oil for cooking. Each participant had a personal interview with a trained dietician and a group session conducted by the same dietician every 3 months during these 4 years	Participants were advised to reduce all types of fat and were given recommendations according to the American guidelines	FFQ	0–14	EGE: 68.1 ± 6.1 EGN: 67.4 ± 5.7 CG: 68.0 ± 6.1	EGE: 91 EGN: 75 CG: 77	/	ELISA kit	Adherence to the MeDi diet was associated to an improvement in plasma BDNF concentrations in individuals with depression and to prevent depression and cognitive decline
Valls-Pedret et al. (66)	Spain	Cognitively normal participants at high CVD risk	4.1 years	Participants were educated on how to follow the MeDi diet and received supplemental foods at no cost. Participants received free allotments of either EVOO (1 L/week) or 30 g/day of raw, unprocessed mixed nuts (15 g walnuts, 7.5 g almonds and 7.5 g hazelnuts)	Participants were advised to reduce all dietary fat	FFQ	0–14	EGE: 67.9 ± 5.4 EGN: 66.7 ± 5.3 CG: 65.5 ± 5.8	EGE: 127 EGN: 112 CG: 95	EGE: 18% EGN: 24% CG: 34%	MMSE, RAVLT, subtest of Wechsler memory scale, animal fluency test, Wechsler adult intelligence scale, CTT	The MeDi diet supplemented with olive oil or nuts was associated with improved cognitive function

MeDi diet, Mediterranean diet; SUCCAB, Swinburne university computerized cognitive assessment battery; CDT, Clock drawing test; MMSE, Mini-Mental State Examination; EVOO, Extra virgin olive oil; EG, Experiment group; CG, Control group; EGE, Mediterranean diet plus Extra virgin olive oil; EGN, Mediterranean diet plus nuts; BDNF, Brain-derived neurotrophic factor; RAVLT, Rey Auditory Verbal Learning Test; CTT, Color trail test.

Five RCTs were eligible for inclusion, all of which had a parallel design and included cognitively stable participants (62–66). Of the five RCTs, two were from Australia with follow-up times of only 0.5 years and an average participant age of over 70 years (62, 63). The other three RCTs were from Spain, with a follow-up duration range of 3–6.5 years and an average participant age of 67 years (65–67). The earliest and most recent trials were conducted in 2011 (65) and 2020 (62), respectively. Of the trials conducted in Spain, intervention groups were subdivided into the following two groups: those receiving a free allotment of extra virgin olive oil (EVOO) or unprocessed mixed nuts. To ensure the accuracy and rigor of the MeDi diet, participants received a collection of recipes or intensive education. As for the control group, participants were asked to maintain their current lifestyle or advised to adhere to a low-fat diet. In the five RCTs, there were 883 participants in the experimental groups and 396 participants in the control groups. Several methods were used to assess cognitive function, including the MMSE, Rey Auditory Verbal Learning Test, and Color Trail Test.

### Quality assessment

Of the 31 cohort studies, 93.6% achieved high-quality NOS scores (*n* = 29), and only two studies had scores of 6 and were, thus, regarded as having a high risk of bias (Supplementary Table 2 shows the NOS grades of the 31 cohort studies). Regarding selection bias, all five RCTs mentioned random sequence generation, and four trials were conducted with allocation concealment (Supplementary Figure 1 summarizes the risk of bias in the five RCTs). All five trials had a high risk of performance bias, without participant blinding, and three trials were blinded to outcome assessment. Four of these trials had a low risk of bias in incomplete outcome data, as it had been properly addressed. One trial, however, had a high risk of attrition bias. All five RCTs were free from reporting and other biases.

### Meta-analysis

#### Effects on cognitive function in cohort studies

**FIGURE 2:**
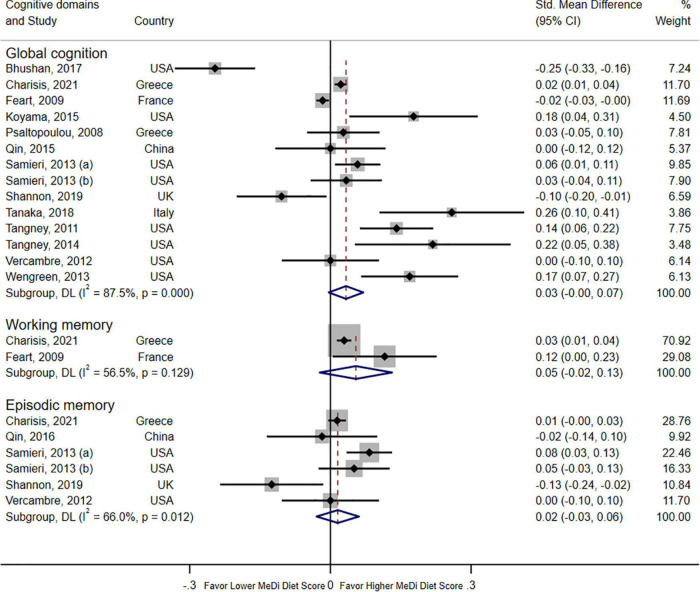
Forest plot for cohort studies with a standardized mean difference (std. mean difference) and 95% CIs showing the associations between the Mediterranean diet score and three domains of cognition function (global cognition, episodic memory, and working memory).

A forest plot revealed the relationship between adherence to the MeDi diet and three domains of cognitive function in 14 cohort studies (Figure 2). Pooled results did not show significant associations with global cognition when compared with the lowest group (SMD = 0.03; 95% CI: 0.00–0.07; I^2^ = 87.5%, *P* < 0.000). No significant associations were found with episodic memory (SMD = 0.02; 95% CI: -0.03–0.06; I^2^ = 66.0%, *P* = 0.012) or working memory (SMD = 0.05; 95% CI: -0.02–0.13; I^2^ = 56.5%, *P* = 0.129).

#### Effects on cognitive disorders in cohort studies

**FIGURE 3:**
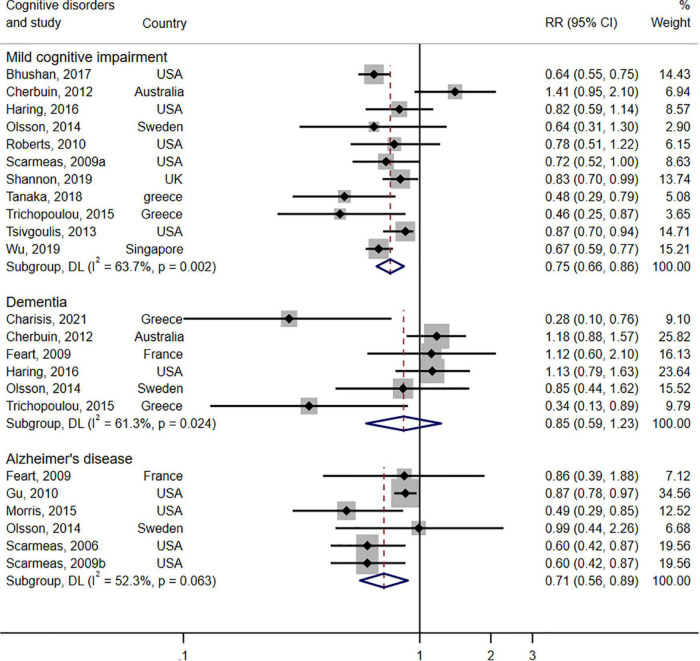
Forest plot for prospective studies of the risk ratio and 95% confidence intervals showing forest plot for the associations between the Mediterranean diet score and the risk of three types of cognitive disorders (mild cognitive impairment, dementia, Alzheimer’s disease).

The forest plot shown in Figure 3 displays the relationship between adherence to the MeDi diet and three types of cognitive disorders in 17 cohort studies. Pooled results of high adherence to the MeDi diet showed a positive association with reduced risk of MCI (RR = 0.75; 95% CI: 0.66–0.86; I^2^ = 63.7%, *P* = 0.002). Moreover, pooled results indicated that high adherence to the MeDi diet was not associated with reduced risk of dementia (RR = 0.85; 95% CI: 0.59–1.23, *P* = 0.399; I^2^ = 61.3%, *P* = 0.024). The pooled analysis, however, indicated that the MeDi diet could reduce the risk of AD by 29% (RR = 0.71; 95% CI: 0.56–0.89; I^2^ = 52.3%, *P* = 0.063).

#### Effects on cognitive function in randomized control trials

**FIGURE 4:**
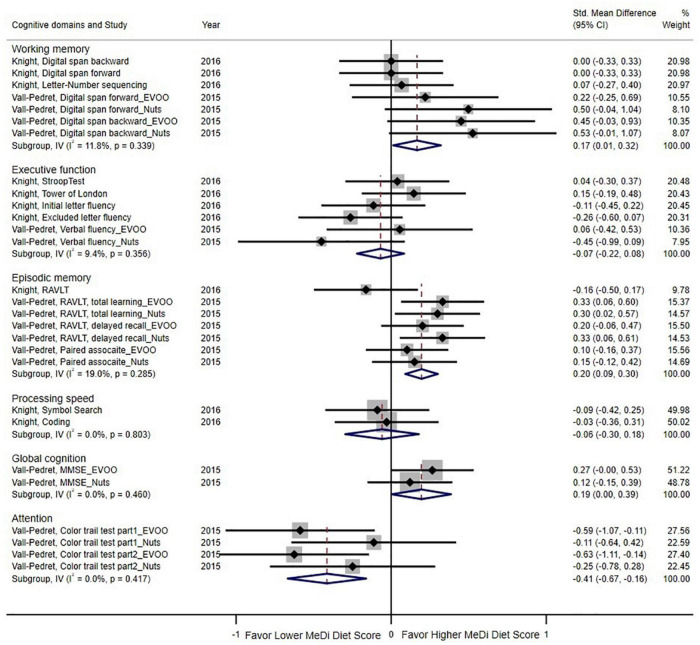
Forest plot for randomized controlled trials of standardized mean difference (std. mean difference) and 95% confidence intervals showing forest plot for the associations between the Mediterranean diet score and cognition function by cognitive domains (working memory, executive function, episodic memory, processing speed, global function, attention). MMSE, Mini-Mental State Examination; EVOO, Extra virgin olive oil; RAVLT, Rey Auditory Verbal Learning Test.

Two RCTs examined the effects on cognitive function based on six types of cognitive domains (Figure 4). The pooled results indicated that the MeDi diet could strengthen working memory (SMD = 0.17; 95% CI: 0.01–0.32, *P* = 0.033; I^2^ = 11.8%, *P* = 0.339), episodic memory (SMD = 0.20; 95% CI: 0.09–0.30, *P < 0.000*; I^2^ = 19.0%, *P* = 0.285), and global cognition (SMD = 0.19; 95% CI: 0.00–0.39, *P* = 0.046; I^2^ = 0.0%, *P* = 0.460) as compared with the control group. On the contrary, the MeDi diet showed an adverse effect on attention (SMD = -0.41; 95% CI: -0.67–0.16, *P* = 0.001; I^2^ = 0.0%, *P* = 0.417) and was not associated with executive function and processing speed (*P* = 0.374, *P* = 0.625, respectively); both parameters showed no between-study heterogeneity (I^2^ = 9.4%, I^2^ = 0.0%, respectively).

### Meta-regression and subgroup analysis

Subgroup and meta-regression analyses were performed in the cohort studies on the following covariates: study location, study published year, duration of follow-up, dietary intake assessment method, and study quality. However, owing to the limited number of studies (<10), the meta-regression was only conducted for global cognition and MCI risk (Supplementary Tables 3, 5).

Regarding the relationship between the MeDi diet and global cognition, no covariates were found to affect heterogeneity except for the dietary assessment method (*P < 0.000*; Supplementary Table 3). A subgroup analysis of different assessment methods showed that studies using methods other than an FFQ had a negative association with global cognition (SMD = -0.02, 95% CI: -0.03–0.00; I^2^ = 0.0%). Pooled results of meta regression analyses revealed that study location, study published year, and duration of follow-up affect heterogeneity (*P* < 0.05) in terms of the MeDi diet and MCI risk, whereas the exposure assessment method and study quality do not affect heterogeneity (*P* = 0.307; *P* = 0.059; Supplementary Table 5). Subgroup analysis found no heterogeneity among Mediterranean region studies (I^2^ = 0.0%), while high adherence to the MeDi diet was associated with lower risk of MCI (RR = 0.57; 95% CI: 0.32–0.70).

The relationship between the MeDi diet and episodic memory or dementia was examined in the subgroup analysis (Supplementary Tables 4, 6). Pooled results revealed a positive association between high MeDi diet score and episodic memory in studies conducted before 2015 with low heterogeneity (SMD = 0.06; 95% CI: 0.02–0.10; I^2^ = 8.4%) and studies with follow up duration < 5 years without heterogeneity (SMD = 0.02; 95% CI: 0.00–0.03; I^2^ = 0.0%; Supplementary Table 4). For dementia, study location, study published year, and exposure assessment method affected heterogeneity. For the Non-Mediterranean region, studies conducted before 2015 and exposure assessments conducted using other method, there was no heterogeneity (All I^2^ = 0.0%; Supplementary Table 6).

### Publication bias

Owing to the limitations in evidence (requiring more than 10 studies), publication bias was only investigated for cohort studies that analyzed the associations between the MeDi diet and global cognition and MCI. No publication bias was found (Supplementary Figure 2 shows the funnel plot), and both Egger’s and Begg’s tests showed no publication bias in the relationship between adherence to the MeDi diet and global cognition in the cohort studies (Egger’s test: *P* = 0.330; Begg’s test: *P* = 0.443). A funnel plot of the relationship between the MeDi diet score and MCI risk in cohort studies was designed (Supplementary Figure 3 shows the funnel plot), and no publication bias was found. Both Egger’s and Begg’s tests showed similar results (Egger’s test: *P* = 0.968; Begg’s test: *P* = 0.876).

## Discussion

This systematic review and meta-analysis qualitatively analyzed 31 cohort studies and five RCTs and quantitatively analyzed 26 cohort studies and two RCTs. Pooled results of the RCTs indicated that adherence to the MeDi diet could increase global cognition, episodic memory, and working memory but may reduce attention. The main findings from the prospective studies indicated that high adherence to the MeDi diet could reduce MCI and AD risks. However, no significant associations between adherence to the MeDi diet and cognitive function or dementia were found in the cohort studies.

Our results on cognitive function from the prospective studies are in partial agreement with those of previous studies. Loughrey et al. conducted the first systematic review that investigated the relationship between the MeDi diet and cognitive function (delayed recall, episodic memory, global memory, and working memory) among healthy older adults (24). Their conclusions were similar to our findings, and the differences were mostly not significant, with a small effect size. However, there was high heterogeneity in the effect size. Several possible explanations were given for the contradiction. First, this could have been due to different MeDi diet scoring methods used in the studies. Similarly, when the same MeDi diet score was used, there were individual differences as it was not possible to ensure that every participant strictly adhered to the MeDi diet. Moreover, the current MeDi diet differs from the traditional MeDi diet owing to social, economic, geographical, cultural, and educational factors (68). In addition, differences in cooking methods may have had an impact on the bioavailability of nutrients which could indirectly affect cognitive function. The impact of cooking method varies. For example, gently fried can enhance glucose metabolism which may be linked to increased cognitive function, and it does not destroy dietary phenolic compounds as compared to frying (69). Studies have found that high-temperature cooking such as frying produces acrylamide, and dietary exposure to acrylamide is associated with cognitive function decline (70). Loughrey et al. inferred that the MeDi diet was beneficial in improving global cognition, which is contrary to our findings (24). This may be due to the use of the MMSE scale in the included studies, which may not be sensitive to cognitive changes in healthy populations, according to Gluhm et al. (71). Secondly, some of the populations included in the studies were relatively young (approximately 40 years). Therefore, the effects of the MeDi diet on cognitive changes may have been highly confounded by other factors.

In 2021, a meta-analysis indicated that the results on the relationship between the MeDi diet and cognitive disorders were similar to those in our study. In particular, high adherence to the MeDi diet was beneficial to lower the risk of MCI and AD (22). The Spanish team included 22 studies in the qualitative analysis and 11 studies in the meta-analysis and concluded that the MeDi diet could lower the risk of MCI (RR = 0.91, 95% CI: 0.85–0.97) and AD (RR = 0.89, 95% CI: 0.84–0.93) (22). Before this, Wu et al. and Singh et al. both reached similar conclusions (20, 21), indicating that higher adherence to the MeDi diet could reduce MCI incidence by 17 and 27%, respectively, and AD incidence by 40 and 36%, respectively. Many meta-analyses have demonstrated the effects of representative food groups or potentially beneficial nutrients in the MeDi diet on cognitive health. The MeDi diet typically includes daily consumption of vegetables, fruits, whole grains, and moderate consumption of fish and red wine, as well as partaking in exercise (8). Intake of foods, such as fruit, vegetables, fish, and cereals, as well as nutrients, including vitamins and omega 3, can reduce mild and even severe cognitive impairment (72–75). The MeDi diet may also reduce cognitive decline by reducing oxidative stress (76, 77). Furthermore, in 2004, Chrysohoou et al. conducted the Attica study and found that adherence to the MeDi diet could lower C-reactive protein (CRP) and interleukin levels, thus protecting cognitive health (78, 79). Gu et al. showed similar results in 2010, indicating that high adherence to the MeDi diet could lower high-sensitivity CRP levels, thereby reducing the risk of AD by 34% (18). Additionally, there is some evidence that olive oil plays a key role in the MeDi diet and may be protective against AD risk, especially in ApoE4 carriers (70, 80). Therefore, it may be important to modulate pathways affected by genetic risk factors (i.e., ApoE 4), as ApoE is the most important susceptibility locus and a non-modifiable genetic risk factor for AD (64, 81). Many systematic reviews uncovered no association between high MeDi diet score and dementia (21, 23). A meta-analysis conducted by Wu et al. indicated that adherence to the MeDi diet was not related to dementia risk (RR = 1.07, 95% CI: 0.81–1.42) (21), which is consistent with our results. A possible explanation for this is that the effect of the MeDi diet on dementia may be in delaying the onset of dementia, which would take at least 5 years, if not 10+ years, to reveal (82).

Regarding the relationship between the MeDi diet and cognitive function in RCTs, the earliest meta-analysis of RCTs on this topic was presented by Loughrey et al. in 2017. They showed that high adherence to the MeDi diet could strengthen delayed recall, global cognition, and working memory, but no such association with attention was found (24). In 2018, Radd-Vagenas et al. also conducted a systematic review and meta-analysis of RCTs and reported the effects of the MeDi diet on seven cognitive domains (global cognition, attention, verbal and visual memory, working memory, processing speed, and executive function) (83). However, part of the results was inconsistent with our conclusion, as we found that high adherence to the MeDi diet had an adverse effect on attention. This discrepancy was likely owing to the limited evidence, as our review only analyzed the RCTs conducted by Vall-Pedret et al. and Knight et al. (63, 66). Therefore, future clinical studies are undoubtedly needed to obtain more convincing results. Secondly, since the Mediterranean diet is a dietary pattern rather than a single diet or diet group, it is possible that one of the diet groups considered as Mediterranean had a negative impact on cognitive function but was masked by the effects of other groups. For example, studies have shown that meat consumption is associated with poorer cognitive function (84). Animal models have revealed that these meat products contain a large amount of saturated fatty acids, trans fatty acids, conjugated linoleic acid, and other substances, which may adversely affect the central nervous system and impact cognitive function (85).

Of note, the pooled results of the included cohort studies and RCTs in our study were not the same, which may have been due to the insufficient number of included studies. Second, differences in the assessment of the MeDi diet also led to differences in the results. Third, the follow-up period was extremely short, as the RCT conducted by Knight et al. lasted for only 6 months (63), while cognitive function needs time to develop detectable change. Lastly, there was a lack of standardized tests for cognitive health to measure changes in cognitive function.

The strength of this paper primarily lies in the following four factors: First, only cohort studies and RCTs were included in this review, and the results were discussed and analyzed separately. Second, this review strictly followed the PRISMA guidelines in the review process, and each step was carefully checked and examined. Third, a meta-regression analysis was conducted to determine the source of heterogeneity. Finally, when performing data extraction, we attempted to contact the authors to obtain accurate raw data. However, there were also limitations to our study. First, the MeDi diet assessment scores and cognitive function testing methods were different, which may have caused bias in the results. Additionally, differences in the dietary assessment methods may also limit comparability and increase error. Third, the follow-up duration of some cohort studies may have been too short to account for changes in cognitive function. Finally, we could not control for dietary differences due to regional variations. Although all studies adhered to the MeDi diet, it was thought that it would be easier and more effective for people from MeDi regions to follow the MeDi diet than for people in other regions.

In summary, this review provided significant evidence that adherence to the MeDi diet could lower the risk of MCI and AD, whereas adherence to the MeDi diet was not related to dementia and other specific cognitive function domains (global cognition, working memory, and episodic memory) in the cohort studies. Across the RCTs, high adherence to the MeDi diet was positively associated with global cognition, working memory, and episodic memory. However, a negative association between the MeDi diet and attention was found. Overall, the MeDi diet is recommended to prevent or delay cognitive disorders and improve cognitive function. These results reinforce further clinical trials on the association between the MeDi diet and cognitive health, with longer follow-up time, especially on attention. Besides that, studies focus on cooking methods, cooking frequency in the MeDi diet was suggested to conduct as well.

## Data availability statement

The original contributions presented in this study are included in the article/Supplementary material, further inquiries can be directed to the corresponding author.

## Author contributions

JF and L-JT: literature review and data extraction, data synthesis and statistical analysis, manuscript drafting. SS and JL: manuscript critical revision. All authors approved the final version to be submitted.
