# Supplementary material for: Association between the mediterranean diet and cognitive health among healthy adults: A systematic review and meta-analysis
Source: Front Nutr. 2022 Jul 28;9:946361. doi: 10.3389/fnut.2022.946361 (PMC9372716; doi:10.3389/fnut.2022.946361)
Supplement: Supplementary file 1 [file Data_Sheet_1.zip › Appendix 2.DOCX]

NEWCASTLE–OTTAWA QUALITY ASSESSMENT SCALE

COHORT STUDIES

Note: A study can be awarded a maximum of 1 star for each numbered item within the selection and outcome categories. A maximum of 2 stars can be given for comparability

**Selection**

1) Representativeness of the exposed cohort

a) truly representative of the underlying population *

b) somewhat representative of the underlying population *

c) selected group of users e.g., nurses, volunteers

d) no description of the derivation of the cohort

2) Selection of the non-exposed cohort

a) drawn from the same community as the exposed cohort *

b) drawn from a different source

c) no description of the derivation of the non-exposed cohort

3) Ascertainment of exposure

a) secure record (e.g. surgical records) *

b) structured interview *

c) written self-report

d) no description

4) Demonstration that the outcome of interest was not present at the start of the study

a) yes *

b) no

**Comparability**

1) Comparability of cohorts on the basis of the design or analysis

a) study controls for main confounders (age, sex, and education) *

b) study controls for any additional factor * (These criteria could be modified to indicate specific control for a second important factor.)

**Outcome**

1) Assessment of outcome

a) independent blind assessment *

b) record linkage *

c) self-report

d) no description

2) Was follow-up long enough for outcomes to occur

a) yes (follow-up > 2 years) *

b) no

3) Adequacy of follow-up of cohorts

a) complete follow-up - all subjects accounted for *

b) subjects lost to follow-up unlikely to introduce bias - small number lost - < 30% (select an adequate %) follow-up, or description provided of those lost*

c) follow-up rate < 70% (select an adequate %) and no description of those lost

d) no statement
