# Supplementary material for: Association between the mediterranean diet and cognitive health among healthy adults: A systematic review and meta-analysis
Source: Front Nutr. 2022 Jul 28;9:946361. doi: 10.3389/fnut.2022.946361 (PMC9372716; doi:10.3389/fnut.2022.946361)

SUPPLEMENTAL DIGITAL CONTENT

**eFigure 1** Risk of bias graph (A) and risk of bias summary (B) in five randomized controlled trials


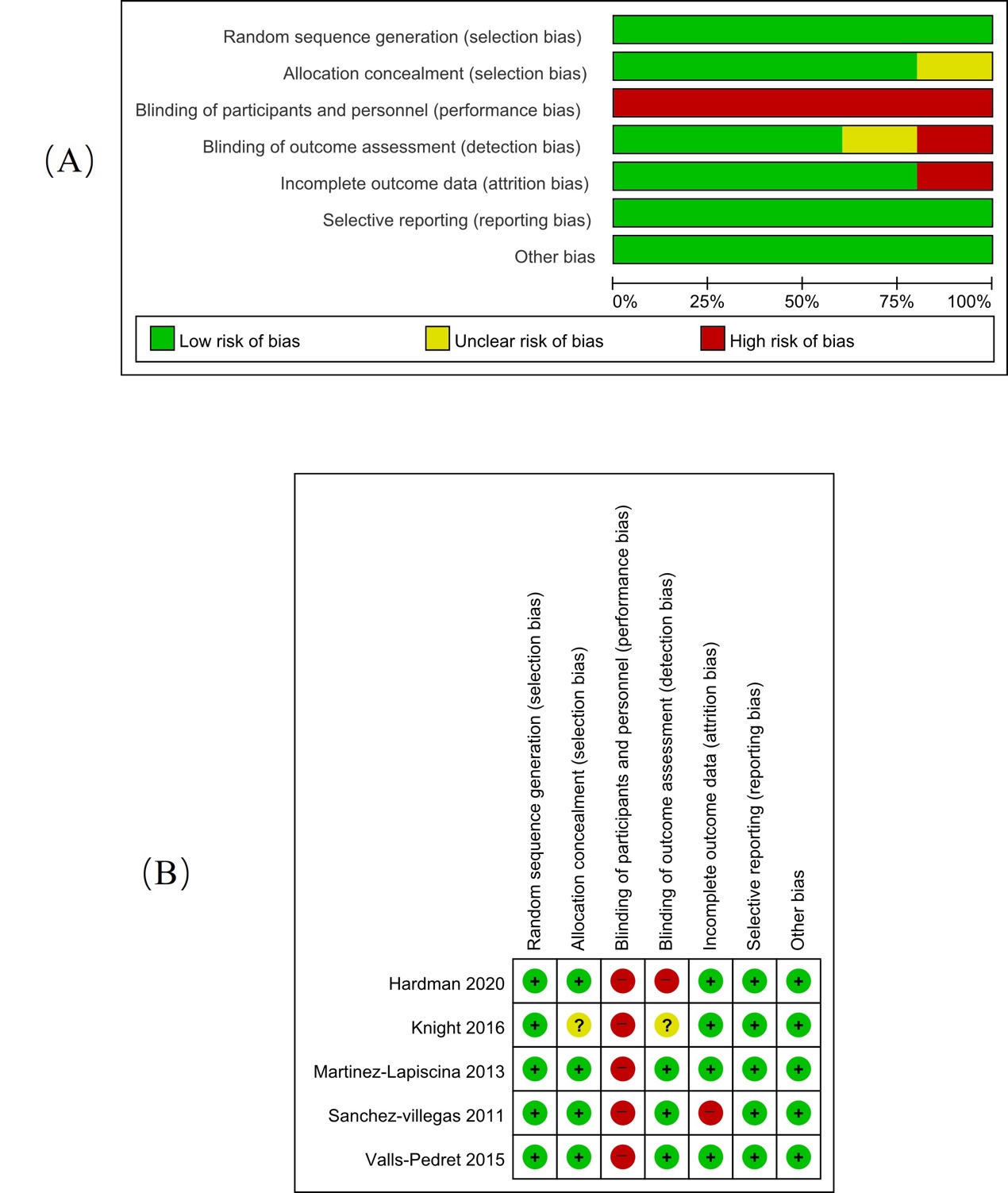


**eFigure 2** Funnel plot of the association between Mediterranean diet score and global cognition in cohort studies. SMD=standard mean difference. (Egger’s test: *P*=.330; Begg’s test: *P*=.443)


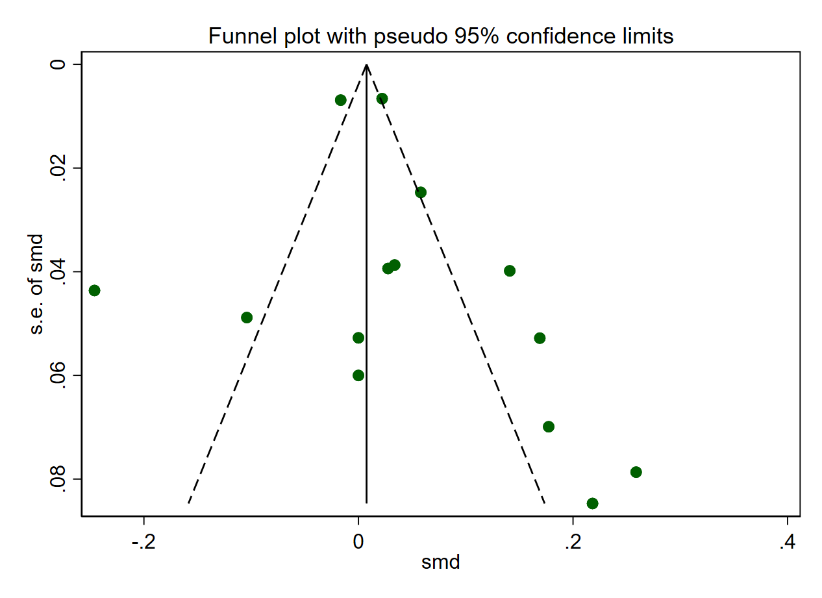


**eFigure 3** Funnel plot of the association between Mediterranean diet score and the risk of mild cognitive impairment in cohort studies. (Egger’s test: *P*=.968; Begg’s test: *P*=.876)


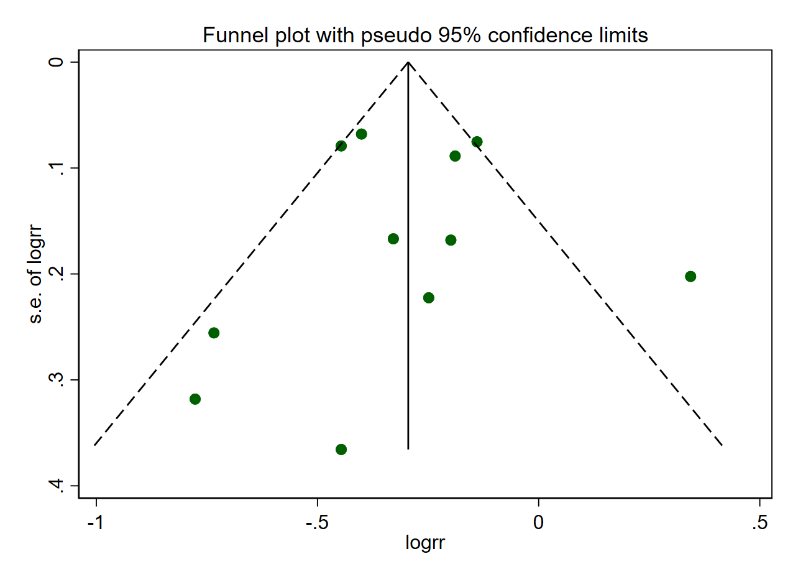

Supplement: Supplementary file 1 [file Data_Sheet_1.zip › Supplementary figures.DOCX]
