# Supplementary material for: Association between the mediterranean diet and cognitive health among healthy adults: A systematic review and meta-analysis
Source: Front Nutr. 2022 Jul 28;9:946361. doi: 10.3389/fnut.2022.946361 (PMC9372716; doi:10.3389/fnut.2022.946361)
Supplement: Supplementary file 1 [file Data_Sheet_1.zip › Supplementary Tables.DOCX]

SUPPLEMENTAL DIGITAL CONTENT

**eTable 1** Electronic search strategy

| **Electronic search strategy** | | |
| --- | --- | --- |
| **Intervention** | **Outcomes** | |
| Mediterranean diet | Cognitive disorders |  |
| MeDi diet | Mild cognitive impairment |  |
| MedDiet | Dementia |  |
| Mediterranean dietary pattern | Alzheimer’s disease |  |
|  | Cognition |  |
|  | Cognitive functions |  |
|  | Attention |  |
|  | Memory |  |
|  | Global cognition |  |
|  | Executive function |  |

We searched five electronic databases: PubMed and Web of Science, PsycINFO, Scopus, Cochrane through the same keywords.

**eTable 2** Quality assessment of the cohort studies: adapted from the Newcastle–Ottawa Scale

| **Author, Published year** | **Selection (maximum 4*)** | **Comparability (maximum 2*)** | **Outcome (maximum 3*)** | **Total score (0–9)** |
| --- | --- | --- | --- | --- |
| Bhushan, 2017 (19) | *** | * | *** | 7 |
| Charisis, 2021 (36) | **** | ** | ** | 8 |
| Cherbuin, 2012 (13) | **** | ** | ** | 8 |
| Feart, 2009 (15) | **** | ** | ** | 8 |
| Galbete, 2015 (39) | **** | ** | *** | 9 |
| Gallucci, 2013 (40) | **** | / | ** | 6 |
| Gardener, 2014 (45) | *** | ** | ** | 7 |
| Gu, 2010 (18) | **** | ** | ** | 8 |
| Haring, 2016 (14) | **** | * | *** | 8 |
| Kesse-Guyot, 2013 (42) | **** | ** | ** | 8 |
| Koyama, 2015 (46) | **** | ** | *** | 9 |
| Lutski, 2020 (41) | ** | ** | ** | 6 |
| Morris, 2015 (17) | *** | ** | ** | 7 |
| Olsson, 2014 (47) | **** | * | *** | 8 |
| Psaltopoulou, 2008 (43) | *** | ** | *** | 8 |
| Qin, 2015 (48) | **** | ** | *** | 9 |
| Roberts, 2010 (49) | **** | ** | ** | 8 |
| Samieri, 2013a (50) | *** | * | *** | 7 |
| Samieri, 2013b (51) | **** | * | *** | 8 |
| Scarmeas, 2009a (52) | **** | ** | ** | 8 |
| Scarmeas, 2009b (53) | **** | ** | ** | 8 |
| Scarmeas, 2006 (54) | **** | ** | ** | 8 |
| Shannon, 2019 (55) | **** | ** | ** | 8 |
| Tanaka, 2018 (37) | **** | ** | ** | 8 |
| Tangney, 2014 (56) | *** | ** | ** | 7 |
| Tangney, 2011 (57) | **** | ** | ** | 8 |
| Trichopoulou, 2015 (38) | *** | ** | ** | 7 |
| Tsivgoulis, 2013 (58) | **** | ** | ** | 8 |
| Vercambre, 2012 (59) | *** | * | *** | 7 |
| Wengreen, 2013 (60)­ | **** | ** | *** | 9 |
| Wu, 2019 (16) | **** | ** | ** | 8 |

Study total scores ≥7 stars are considered as high quality, study total scores 5–6 stars are considered as medium quality, study total scores <5 stars are considered as low quality

**eTable 3** Subgroup and meta–regression analyses of the association between the Mediterranean diet score and global cognition in cohort studies

| Subgroup | Heterogeneity | | | | Meta–regression | | | | |
| --- | --- | --- | --- | --- | --- | --- | --- | --- | --- |
|  | Studies, No. | SMD (95% CI) | I^2^，% | P value* | Coefficient | Std. err. | Z | P value | 95%CI |
| Study location | 14 |  |  | 0.075 | 0.02 | 0.07 | 0.27 | 0.789 | -0.12,0.16 |
| Mediterranean region | 4 | 0.00 (0.00,0.01) | 89.0% |  |  |  |  |  |  |
| Non–Mediterranean region | 10 | 0.03 (0.00,0.06) | 87.8% |  |  |  |  |  |  |
| Study published year | 14 |  |  | 0.044 | -0.07 | 0.06 | -1.09 | 0.275 | -0.19,0.05 |
| ≥2015 | 6 | 0.02(0.00,0.03) | 91.4% |  |  |  |  |  |  |
| <2015 | 8 | 0.00(-0.01,0.01) | 83.3% |  |  |  |  |  |  |
| Duration of follow–up (years) | 14 |  |  | 0.023 | 0.04 | 0.07 | 0.56 | 0.576 | -0.10,0.19 |
| ≥5 | 11 | 0.04(0.01,0.06) | 87.8% |  |  |  |  |  |  |
| <5 | 3 | 0.00(-0.01,0.01) | 88.2% |  |  |  |  |  |  |
| Exposure assessment method | 14 |  |  | 0.000 | 0.04 | 0.01 | 4.61 | 0.000 | 0.02,0.06 |
| FFQ | 12 | 0.03(0.01,0.04) | 86.7% |  |  |  |  |  |  |
| Other method | 2 | -0.02(-0.03,0.00) | 0.0% |  |  |  |  |  |  |
| Study quality | 14 |  |  | 0.007 | 0.06 | 0.04 | 1.37 | 0.171 | -0.03,0.15 |
| Scores =9 | 3 | 0.12(0.05,0.18) | 63.7% |  |  |  |  |  |  |
| Scores =8 | ~~8~~ 7 | 0.01(0.00,0.02) | 86.5% |  |  |  |  |  |  |
| Scores =7 | 4 | 0.00(-0.04,0.04) | 93.2% |  |  |  |  |  |  |

**P* value for heterogeneity between groups; SMD: Standard mean deviation; FFQ: Food frequency quest­­­ionnaire; Std. err.: Standard error

**eTable 4** Subgroup analysis of the association between the Mediterranean diet score and episodic memory in cohort studies

| Subgroup | Heterogeneity | | | |
| --- | --- | --- | --- | --- |
|  | Studies, No. | SMD (95% CI) | I^2^，% | P value* |
| Study location | 6 |  |  | 0.877 |
| Mediterranean region | 1 | 0.01(0.00, 0.03) | / |  |
| Non–Mediterranean region | 5 | 0.01(-0.06,0.08) | 69.8% |  |
| Study published year | 6 |  |  | 0.050 |
| ≥2015 | 3 | -0.03(-0.11,0.05) | 67.8% |  |
| <2015 | 3 | 0.06(0.02,0.10) | 8.4% |  |
| Duration of follow–up (years) | 6 |  |  | 0.631 |
| ≥5 | 4 | -0.01(-0.10,0.09) | 77.2% |  |
| <5 | 2 | 0.02(0.00,0.03) | 0.0% |  |
| Exposure assessment method | 6 |  |  | 0.578 |
| FFQ | 5 | 0.02(-0.03,0.07) | 72.0% |  |
| Other method | 1 | -0.02(-0.14, 0.10) | / |  |
| Study quality | 6 |  |  | 0.442 |
| Scores =9 | 1 | -0.02(-0.14, 0.10) | / |  |
| Scores =8 | 3 | -0.01(-0.08,0.07) | 71.5% |  |
| Scores =7 | 2 | 0.05(-0.02, 0.13) | 50.7% |  |

**P* value for heterogeneity between groups; SMD: Standard mean deviation; FFQ: Food frequency questionnaire

**eTable 5** Subgroup and meta–regression analyses of the association between the Mediterranean diet score and the risk of mild cognitive impairment in cohort studies

| Subgroup | Heterogeneity | | | | Meta–regression | | | | |
| --- | --- | --- | --- | --- | --- | --- | --- | --- | --- |
|  | Studies, No. | RR (95% CI) | I^2^，% | P value* | Coefficient | Std. err. | Z | P value | 95%CI |
| Study location | 11 |  |  | 0.016 | 0.50 | 0.23 | 2.21 | 0.027 | 0.06,0.94 |
| Mediterranean region | 2 | 0.47(0.32,0.70) | 0.0% |  |  |  |  |  |  |
| Non–Mediterranean region | 9 | 0.78(0.69,0.89) | 63.9% |  |  |  |  |  |  |
| Study published year | 11 |  |  | 0.069 | 0.24 | 0.09 | 2.58 | 0.010 | 0.06,0.41 |
| ≥2015 | 6 | 0.69(0.60,0.79) | 50.1% |  |  |  |  |  |  |
| <2015 | 5 | 0.87(0.70,1.09) | 49.5% |  |  |  |  |  |  |
| Duration of follow–up (years) | 11 |  |  | 0.052 | 0.25 | 0.09 | 2.71 | 0.007 | 0.07,0.42 |
| ≥5 | 7 | 0.69(0.61,0.78) | 40.3% |  |  |  |  |  |  |
| <5 | 4 | 0.90(0.71,1.14) | 58.3% |  |  |  |  |  |  |
| Exposure assessment method | 11 |  |  | 0.186 | -0.15 | 0.15 | -1.02 | 0.307 | -0.43,0.14 |
| FFQ | 9 | 0.77(0.66,0.90) | 66.7% |  |  |  |  |  |  |
| Other method | 2 | 0.67(0.59,0.76) | 0.0% |  |  |  |  |  |  |
| Study quality | 11 |  |  | 0.036 | -0.24 | 0.13 | -1.89 | 0.059 | -0.51,0.01 |
| Scores =8 | 9 | 0.79(0.68,0.91) | 60.7% |  |  |  |  |  |  |
| Scores =7 | 2 | 0.63(0.53,0.73) | 1.5% |  |  |  |  |  |  |

* *P* value for heterogeneity between groups; RR: Risk ratios; FFQ: Food frequency questionnaire; Std. err.: Standard error

**eTable 6** Subgroup analysis of the association between the Mediterranean diet score and the risk of dementia in cohort studies

| Subgroup | Heterogeneity | | | |
| --- | --- | --- | --- | --- |
|  | Studies, No. | RR (95% CI) | I^2^，% | P value* |
| Study location | 6 |  |  | 0.109 |
| Mediterranean region | 3 | 0.51(0.20,1.31) | 72.5% |  |
| Non–Mediterranean region | 3 | 1.12(0.91,1.39) | 0.0% |  |
| Study published year | 6 |  |  | 0.149 |
| ≥2015 | 3 | 0.52(0.19,1.43) | 80.9% |  |
| <2015 | 3 | 1.12(0.88,1.43) | 0.0% |  |
| Duration of follow–up (years) | 6 |  |  | 0.887 |
| ≥5 | 3 | 0.79(0.44,1.42) | 62.8% |  |
| <5 | 3 | 0.84(0.43,1.62) | 72.1% |  |
| Exposure assessment method | 6 |  |  | 0.439 |
| FFQ | 4 | 0.74(0.43,1.28) | 76.1% |  |
| Other method | 2 | 0.98(0.62,1.54) | 0.0% |  |
| Study quality | 6 |  |  | 0.040 |
| Scores =8 | 5 | 0.98(0.72,1.34) | 48.3% |  |
| Scores =7 | 1 | 0.34(0.13,0.89) | / |  |

**P* value for heterogeneity between groups; RR: Risk ratios; FFQ: Food frequency questionnaire
